# Asymmetry and evolution over a one-year period of the upward rotation of the scapula in youth baseball pitchers

**DOI:** 10.1080/23335432.2018.1499441

**Published:** 2018-09-10

**Authors:** Erik van der Graaff, Bengt Kom, Femke van Dis, Xavier Gasparutto, Marco Hoozemans, Dirkjan Veeger

**Affiliations:** a Department of Human Movement Sciences, Faculty of Behavioral and Movement Sciences, Vrije Universiteit Amsterdam, Amsterdam Movement Sciences, Amsterdam, Netherlands; b Deparment of Biomechanical Engineering, Faculty Mechanical, Maritime and Materials Engineering, Delft University of Technology, Delft, Netherlands

**Keywords:** Pitching, baseball, overhead throwing, scapular upward rotation, scapular dyskinesis

## Abstract

The pitching motion is an asymmetric action by which coordination of scapular rotation in the dominant arm might be affected in time and in comparison with the non-dominant arm. The study aimed to compare asymmetry and the evolution of scapular upward rotation over a one-year period. Data were collected twice, before and after a one-year period, from 92 participants (age = 15.1 SD 1.4 years, body height = 177.3 SD 10.9 cm, body weight 69.2 SD 14.5 kg). Scapular motion was tracked at different glenohumeral angles of elevation in the scapular plane: anatomical position (0°), 45°, 90° and 135°. Scapular upward rotation was calculated as the angle between the spinae scapula and the spine. Scapular upward rotation of the dominant arm was 5.1° (95% CI: 2.1°−8.1°) more compared to the non-dominant arm. Age group or glenohumeral angles of elevation did not affect this difference. Scapular upward rotation of the dominant arm decreased 1.9° (95% CI: −0.5° to 4.3°) after a one-year period, however, neither this observation, nor the interaction with age group or elevation angle was significant. These findings may indicate that pitchers could be at risk to develop shoulder injuries especially those that have been associated with scapular asymmetry.

## Introduction

Overhead throwing athletes solve a complex full body problem in order to generate high throwing velocities. Baseball (170 km/h), handball (130 km/h) and javelin (113 km/h) are all examples of sports that involve very high throwing velocities. Such high speeds can only be reached with a perfected technique that involves the entire body (Putnam 1993; Matsuo et al. 2001). On of the components of this throwing action, the scapula, plays an important role in controlling the shoulder joint. The scapula must move in accordance with the humerus to provide a stable base for the humeral head, but also plays an important role in the transfer of energy from torso to arm (Borsa et al. 2008; Forthomme et al. 2008). Furthermore, the scapula provides congruence between the humeral head and the glenoid cavity to stabilize the glenohumeral (GH) joint (Borsa et al. 2003; Kibler and Sciascia 2010). In sum, scapular support is essential for stability and mobility during the throwing action in overhead throwing athletes.

It is often considered problematic when differences in the kinematics between the left and right scapula are found in athletes performing overhead throws, even though in many asymptomatic athletes such asymmetry exists (Oyama et al. 2008). Thomas et al. (2010) concluded that the asymmetric passive range of motion in combination with scapular dyskinesis might predispose to injury. Dyskinesis is defined as an asymmetric scapular movement due to physiological constraints in the shoulder girdle (Kibler et al. 2012). Asymptomatic throwing athletes have been reported to have several adaptations in their dominant shoulders during humeral elevation tasks. However, there is no clear evidence of a causal relationship between altered scapular kinematics and shoulder injury (Downar et al. 2005).

Upward scapular rotation is one of the constituent movement directions of the scapula and during humeral elevation tasks it does rotate approximately 60° relative to the thoracic cage (McClure et al. 2001). Upward rotation is important during overhead activity to prevent impingement of the rotator cuff (Downar et al. 2005); it prevents the humeral head from compressing against the acromion and thus creating a narrow subacromial space (Myers et al. 2005). Decreased upward rotation has been found to have a high correlation with shoulder injury (Kibler 1998; Ludewig and Cook 2000; Borsa et al. 2003; Burkhart et al. 2003; Oyama et al. 2008). Both Borsa et al. (2008) and Myers et al. (2005) reported increased upward rotation of the scapula in healthy overhead throwing athletes compared to non-throwing athletes. In addition, a significantly larger amount of scapular upward rotation compared to their non-dominant shoulder was observed in the dominant throwing arm of professional baseball pitchers (age 20 SD 1.6 years) with no previous history of shoulder injury (Downar et al. 2005; Laudner et al. 2007). Furthermore, in populations of pitchers aged between 10 and 20 years of age, scapular upward rotation has been found to decrease with age (Mourtacos et al. 2003; Thomas et al. 2010). However, these studies with a cross sectional design that did not address the possible decrease of upward rotation occurring over time.

The aim of the present study was to examine the asymmetry in and the development of scapular upward rotation over a one-year period within a group of elite youth baseball pitchers. To this end, it was examined whether scapular upward rotation is different between the dominant (throwing) arm and the non-dominant arm, whether scapular upward rotation of the dominant arm changes over a one-year period, and whether these potential effects are different for players of different ages. It was hypothesized that pitchers will demonstrate a greater upward rotation in the dominant arm than in the non-dominant arm and that the asymmetry decreases with age.

## Method

### Participants and study design

Data were collected from 92 male baseball pitchers (mean age = 15.1 SD 1.4 years, mean body height = 177.3 SD 10.9 cm, mean body weight = 69.2 SD 14.5 kg), who were playing in the Dutch baseball academies and the national youth (U18) baseball team. Participants were tested twice. The pre-test was conducted in April 2014 and the post-test in March 2015. A division in two age groups was made, based on the team division in the local baseball competition, a younger (*n* = 56, age = 13.8 SD 0.99 years) and an older (*n* = 36, age = 16.6 SD 0.92 years) group. The research design and protocol were approved by the local ethical committee of the Department of Human Movement Sciences before its conductance. Informed consent was obtained from the parents of the participants before participating in the study.

### Procedure

**Figure 1.**
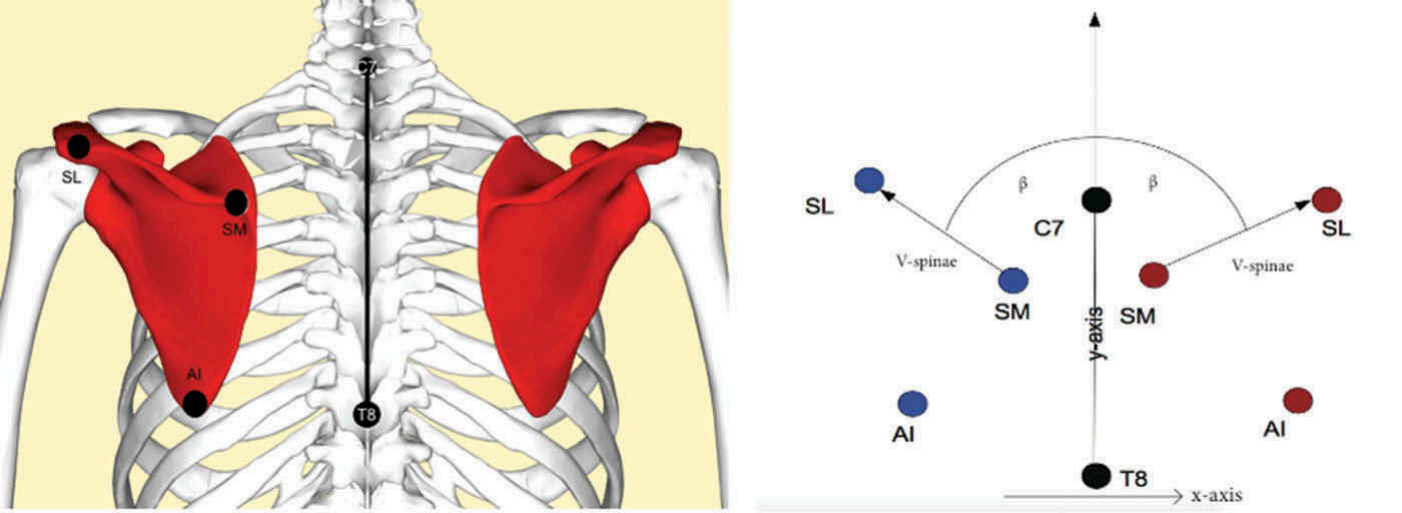
(a) Anatomical landmarks C7, T8, SL (Spina Scapulae Lateralis), SM (Spina Scapulae Medialis) and AI (Angulus Inferior), and (b) explanation of angle *β*, which was calculated using vectors SM-SL and T8-C7 (see Equations 1–4)

**Figure 2.**
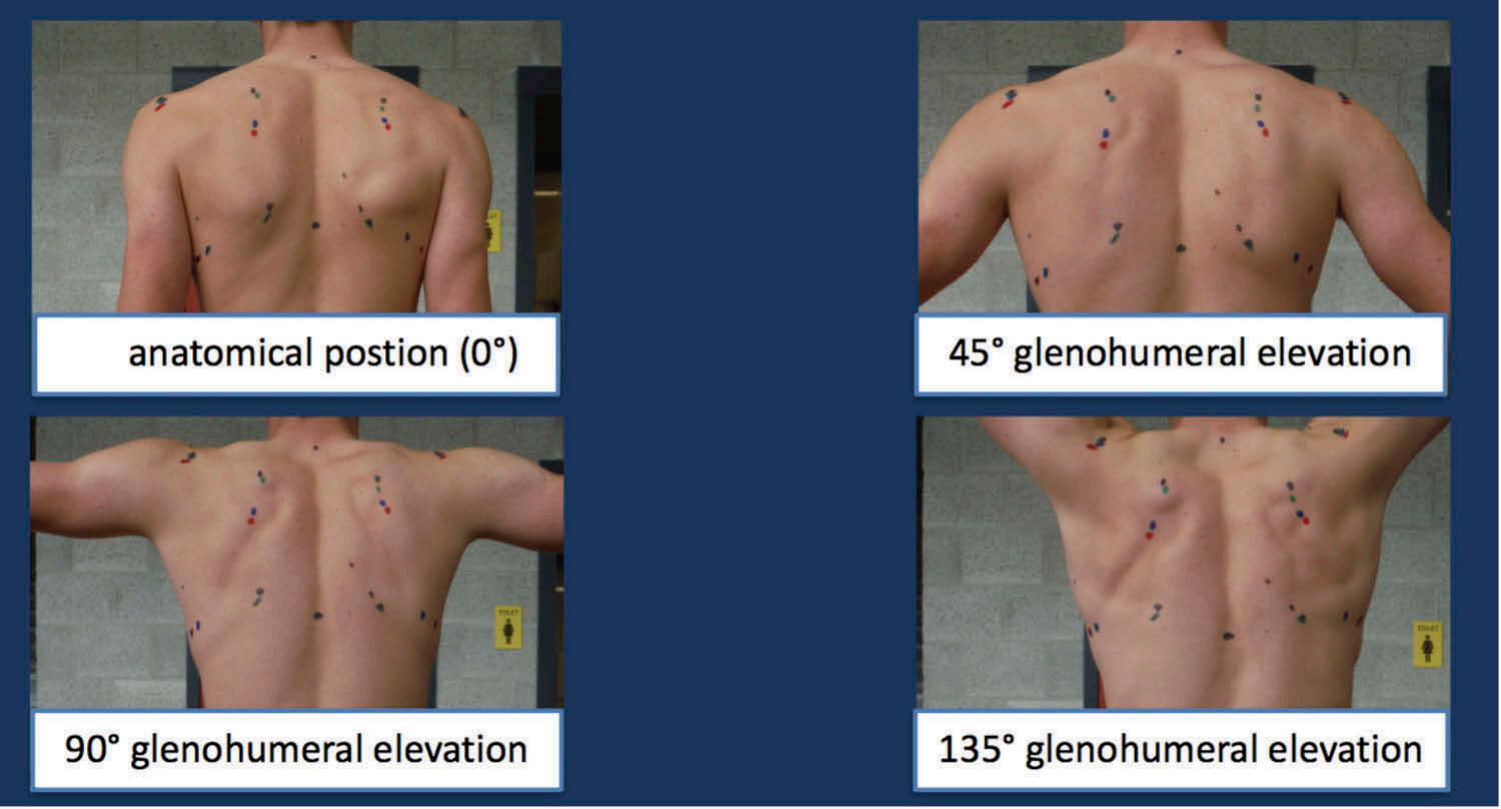
Example of the four positions in which anatomical landmarks were palpated and marked. Results of the palpation were marked with colored permanent markers: black 0°, green 45°, blue 90° and red 135°

The trigonum spinae (SM), angulus acromialis (SL) and the angulus inferior (AI) (Figure 1) of the scapula were palpated and marked with colored permanent markers on the skin at four different GH angles of elevation in the scapular plane: anatomical position (0°), 45°, 90° and 135° (Figure 2). No specific warm-up was performed before palpation. All tests were performed after school hours (as replacement of a regular training). A trained and experienced physiotherapist conducted all palpations. Corresponding to the bony landmarks, SM, SL and AI were located and their coordinates identified. The angle of the scapula with respect to the spine was determined with these coordinates using custom Matlab programming (The MathWorks, Inc, Natick, MA, USA). The spine, which served as the *y*-axis, was defined as the vector from the marker at cervical vertebrae 7 (C7) and at thoracic vertebrae 8 (T8) and normed to length one (Equation 1).

(1) $$Y\left(axis\right)=\;\frac{\left[\begin{array}{cc} X\left(C7\right) & Y\left(C7\right) \end{array}\right]-\left[\begin{array}{cc} X\left(T8\right) & Y\left(T8\right) \end{array}\right]}{X\left(C7\right)\;Y\left(C7\right)-\left|\left|X\left(T8\right)\;Y\left(T8\right)\right|\right|}$$

The x-axis was defined perpendicular to the *y*-axis (Equation 2).

(2) $$X\left(axis\right)=\;\left[\begin{array}{cc} Y\left(y\right) & Y\left(-x\right) \end{array}\right]$$

The scapula was Ↄ defined as a vector (*V_spinae_*) from SM to SL (Equation 3).

(3) $$V_{spinae}=\;\left[\begin{array}{cc} X\left(sl\right) & Y\left(sl\right) \end{array}\right]-\left[\begin{array}{cc} X\left(sm\right) & Y\left(sm\right) \end{array}\right]$$

The angle *β* was calculated as the angle between the vector of the *y*-axis and the vector *V_spinae_* (Equation 4).

(4) $$\beta =cos^{-1}\left(\frac{X\left(axis\right)*\;V_{spinae}}{X\left(axis\right)*V_{spinae}}\right)$$

The upward rotation was calculated as 90° – *β*. In this way, when the spinae scapula was horizontal, the angle for upward rotation was 0°.

### Statistical analysis

To determine whether the amount of scapular upward rotation (β; dependent variable) was different between the dominant and non-dominant arm and whether these differences were affected by the level of arm elevation (0°, 45°, 90°, 135°) and age group (young and old), data from the pre-test were analyzed statistically using a three-way mixed design ANOVA. A three-way mixed design ANOVA was also used to examine whether the scapular upward rotation of the dominant arm was different between the pre-test and post-test and whether these differences were affected by the level of arm elevation and age group. One-way within- and between-subjects ANOVAs with Bonferroni correction were used to examine if the interaction effects were significant. The assumption of normality was checked by means of visual inspection of the histogram, q-q plot and the box plot of the data within the groups. *Z*-values of skewness and kurtosis, and a Shapiro-Wilks test were also performed on the data. Homogeneity of variance was checked using Levene’s test. There were no violations of these assumptions. All statistical analyses were performed in SPSS v23.0.0.2 (IBM Corporation, Armonk, NY, USA) and a *p*-value below 0.05 was considered significant.

## Results

### Asymmetry in scapular upward rotation

**Figure 3.**
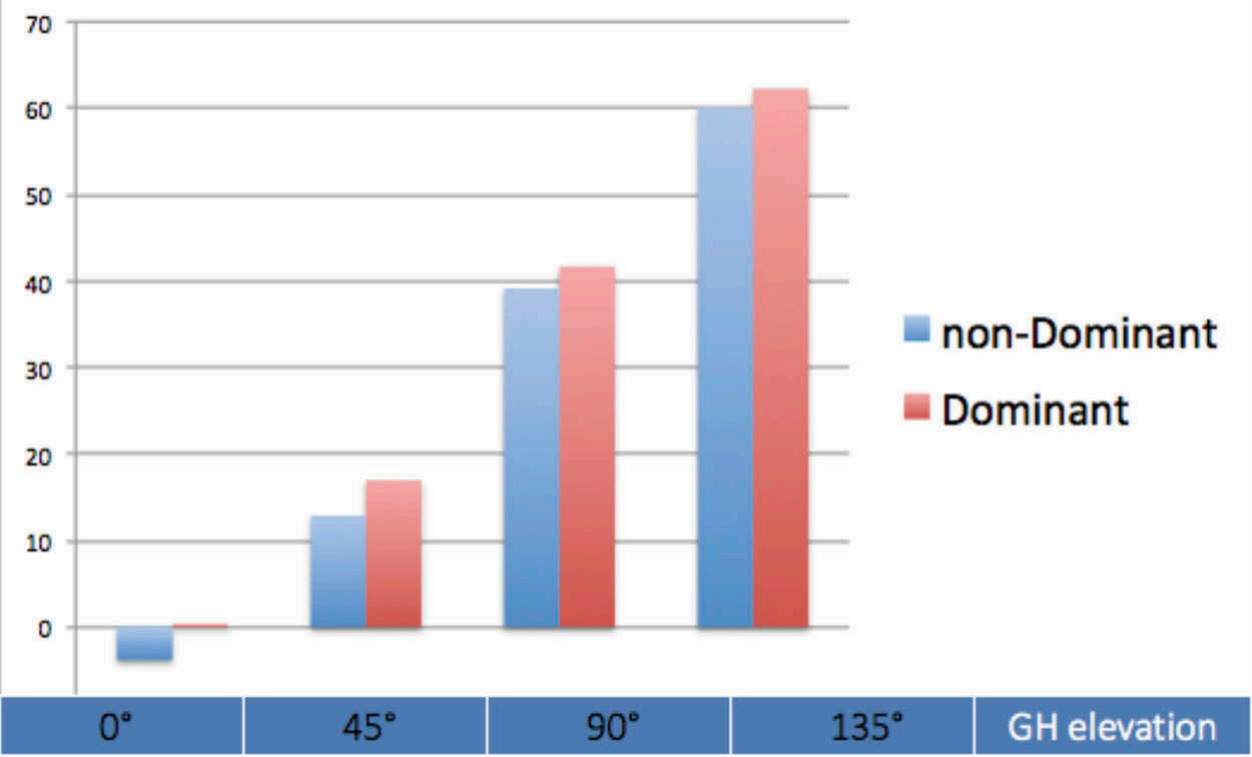
Upward scapular rotation for the dominant (throwing) arm and non-dominant arm for the different GH-elevation angles

Asymmetry between the dominant and non-dominant arm was 5.1° on average (95% CI: 2.1°–8.1°, *F*(1,56) = 11.36, *p* < 0.001), with the dominant arm showing more upward rotation than the non-dominant arm. There was no significant interaction effect of age group (*F*(1,57) = 1.13, *p* = 0.293) or angle of arm elevation (*F*(3,171) = 1.43, *p* = 0.235) on the asymmetry. Dominant and non-dominant scapular upward rotation for all studied angles of arm elevation is displayed in Figure 3.

### Change in scapular upward rotation of the dominant arm

**Figure 4.**
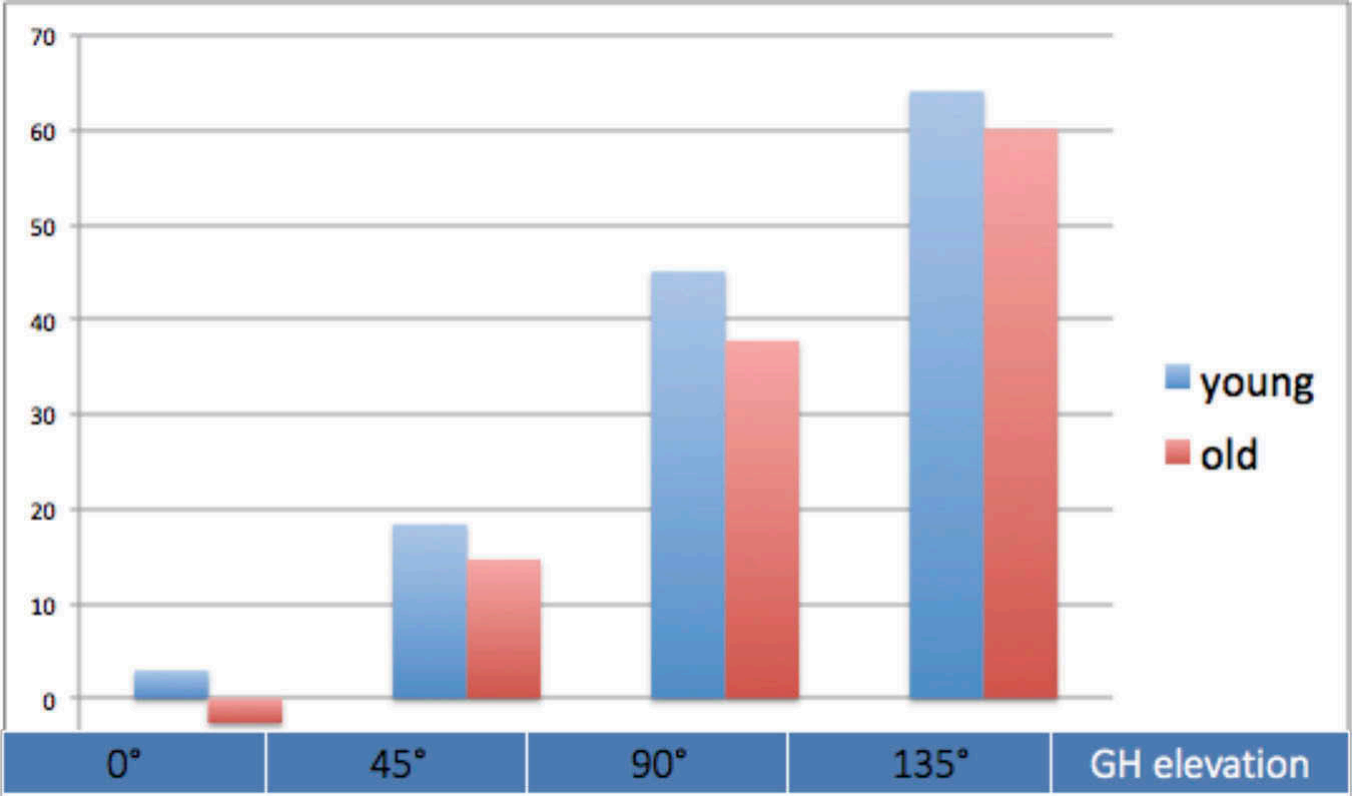
Upward rotation of dominant scapula for the young and old age groups and for the different GH-elevation angles

**Figure 5.**
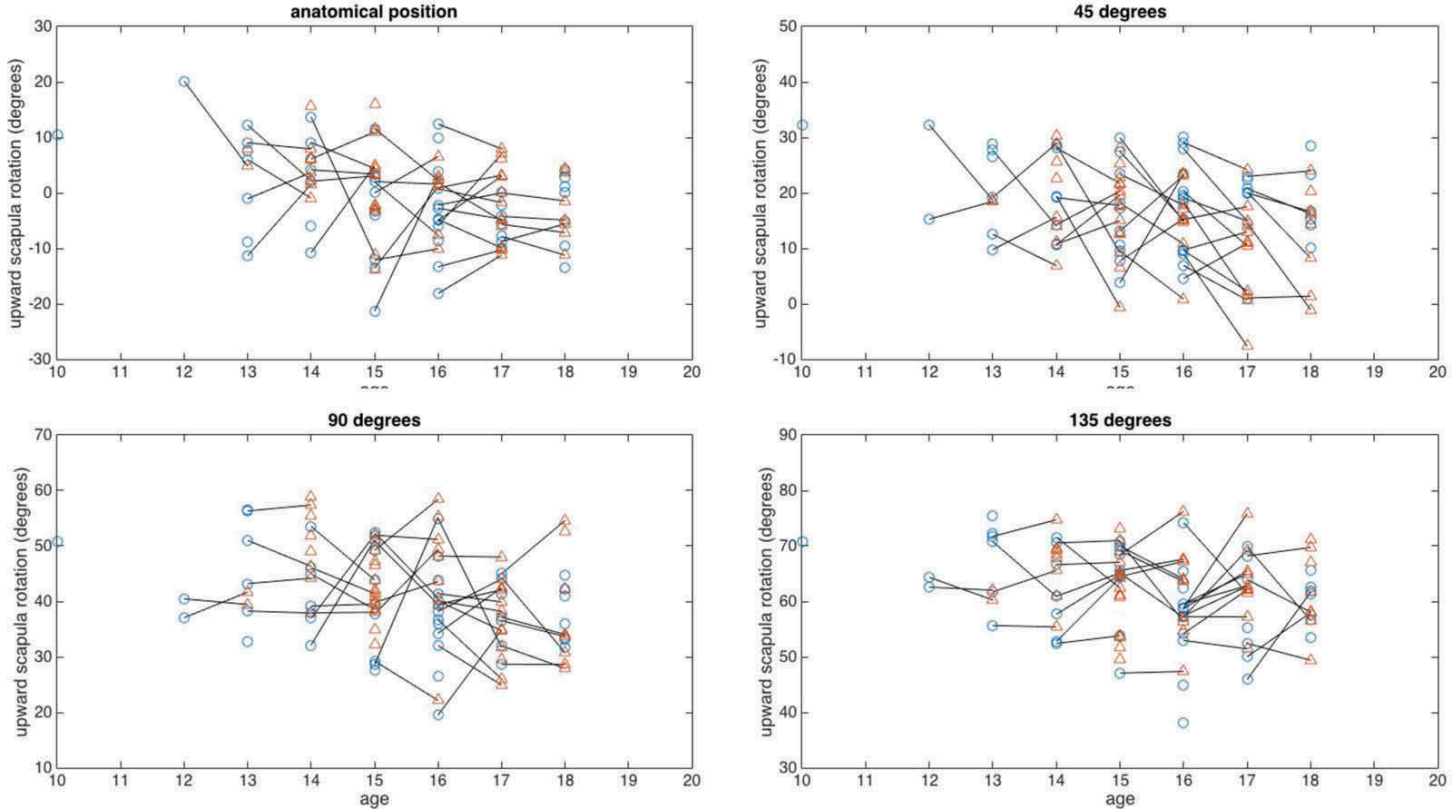
Development of upward rotation between pre-(Ο) and post(Δ) testing. Lines connect results per player

The scapular upward rotation of the dominant arm decreased with 1.9° on average over the one-year period, but this change was non-significant (95% CI: −0.5° to 4.3°, *F*(1,37) = 2.53, *p* = 0.120). The scapular upward rotation of the dominant arm is shown for both age groups and the four angles of GH-elevation in Figure 4. Age group did not significantly affect the change in scapular upward rotation during the one-year period (*F*(1,37) = 1.53, *p* = 0.224). Also, the interaction between time (pre-test/post-test) and angle of arm elevation for scapular upward rotation was not significant (*F*(1.95,72.18) = 1.58, *p* = 0.213) (Figure 5).

## Discussion

As hypothesized, an asymmetry was observed in scapular upward rotation between the dominant and non-dominant shoulder in elite youth baseball pitchers. However, no significant differences were observed between the group of 12–15 years of age and the group of 16–19 years or for different angles of GH-elevation. Also no significant evolution of the scapular upward rotation was observed over the one-year study period.

### Asymmetry in scapular upward rotation

The magnitude of asymmetry in upward rotation observed in the present study (5.10° on average) is similar to that in a couple of other studies (2.1° (Thomas et al. 2010), 3.1° (Mourtacos et al. 2003), and 3.6° (Downar et al. (2005), the latter for 90° arm elevation only). In the present study, asymmetry in upward rotation was observed for all angles of GH-elevation in the absence of a significant interaction effect between arm elevation angle and upward rotation. The present study aimed to cast light on the evolution of this asymmetry as well. However, no significant difference in asymmetry between age groups was observed, although such a cross-sectional difference has been reported in other studies (Tsai et al. 2003; Thomas et al. 2010). A factor contributing to the difference between age groups could be a proportionate increase in muscle strength of the older age group due to the higher amount of testosterone in older age groups (17–18 years old) compared to younger age groups (11–12 and 13–14 years old) (Ramos et al. 1998). For the elite youth pitchers of the present study, it could be considered that the main factor contributing to the asymmetry is that pitchers only throw with their dominant arm. This was the case for both age groups (12–15 years and 16–19 years) and differences in muscle growth between these age groups apparently not cause differences in asymmetry in upward rotation. However, the method used to assess scapular upward rotation yielded quasi-static measurements, which represents a limitation; the exact position of the scapula can be different in upward or downward movement of the arm, especially when a pitcher has less scapular control. Also, our marker method is less accurate than, for instance, X-ray measurements, but the palpation was conducted by a trained and experienced physiotherapist.

### Change in scapular upward rotation of the dominant arm

The present study demonstrated a non-significant decrease in the scapular upward rotation in youth baseball pitchers of 1.9° over the one-year study period (95% CI: −0.5° to 4.3°). A decrease in scapular upward rotation in the 16–19 years old age group was expected because an increase of the posterior capsule thickness of the dominant arm has a positive relationship with upward scapular rotation, leading to fatigue-related inhibition of the scapular muscles on the long term (Thomas et al. 2010). However, the mean decrease in scapular upward rotation observed over the one-year study period was not significant and there was no significant interaction effect for age group, suggesting that both age groups demonstrated comparable changes in scapular upward rotation. Nevertheless, the results of the present study might be clinically important. Apparently, the older participants in this study of youth baseball pitcher exhibited not more scapular upward rotation compared to the younger participants and also no significant increase was observed within both age groups over a year. It can generally be assumed, however, that the physical strength increases of these young pitchers increases as they grow older, and therefore throw faster. As several studies indicated that a decrease in scapular upward rotation may be associated with an increase in (shoulder) injury risk (Kibler 1998; Ludewig and Cook 2000; Borsa et al. 2003; Burkhart et al. 2003; Oyama et al. 2008), monitoring these – and other – young elite pitchers in their physical development, for instance for scapular upward rotation, and their performance (throwing speed) might be important in the prevention of pitching-related shoulder symptoms. Thus, future research should focus on assessing scapular motion over a longer time period during the development of young athletes, especially in asymmetric, injury prone sports, such as baseball pitching.

## Conclusion

This study showed that the dominant arms of elite youth baseball pitchers exhibit more scapular upward rotation compared to their non-dominant arm. However, scapular upward rotation was not greater for the older pitchers as compared to the younger pitcher and it also did not increase over the one-year study period in either age group. As a result of the asymmetry in upward scapular rotation, these athletes could be at risk to develop shoulder injuries, and thus in the training program of these athletes specific exercises could be incorporated to minimize scapular upward rotation of the throwing arm.

## References

[cit0001] Borsa PA, Laudner KG, Sauers EL. 2008. Mobility and stability adaptations in the shoulder of the overhead athlete. Sports Med. 38(1):17–36. 18081365 10.2165/00007256-200838010-00003

[cit0002] Borsa PA, Timmons MK, Sauers EL. 2003. Scapular-positioning patterns during humeral elevation in unimpaired shoulders. J Athl Train. 38(1):12. 12937466 PMC155505

[cit0003] Burkhart SS, Morgan CD, Kibler WB. 2003. The disabled throwing shoulder: spectrum of pathology Part III: the SICK scapula, scapular dyskinesis, the kinetic chain, and rehabilitation. J Arthroscopic Relat Surg. 19(6):641–661. 10.1016/s0749-8063(03)00389-x 12861203

[cit0004] Downar JM, Sauers EL. 2005. Clinical measures of shoulder mobility in the professional baseball player. J Athl Train. 40(1):23. 15902320 PMC1088341

[cit0005] Forthomme B, Crielaard JM, Croisier JL. 2008. Scapular positioning in athlete’s shoulder. Sports Med. 38(5):369–386. 18416592 10.2165/00007256-200838050-00002

[cit0006] Kibler WB. 1998. The role of the scapula in athletic shoulder function. Am J Sports Med. 26(2):325–337. 9548131 10.1177/03635465980260022801

[cit0007] Kibler WB, Sciascia A. 2010. Current concepts: scapular dyskinesis. Br J Sports Med. 44(5):300–305. 19996329 10.1136/bjsm.2009.058834

[cit0008] Kibler WB, Sciascia A, Wilkes T. 2012. Scapular dyskinesis and its relation to shoulder injury. J Am Acad Orthopaedic Surgeons. 20(6):364–372. 10.5435/JAAOS-20-06-364 22661566

[cit0009] Laudner KG, Stanek JM, Meister K. 2007. Differences in scapular upward rotation between baseball pitchers and position players. Am J Sports Med. 35(12):2091–2095. 17687122 10.1177/0363546507305098

[cit0010] Ludewig PM, Cook TM. 2000. Alterations in shoulder kinematics and associated muscle activity in people with symptoms of shoulder impingement. Phys Ther. 80(3):276–291. 10696154

[cit0011] Matsuo T, Escamilla RF, Fleisig GS, Barrentine SW, Andrews JR. 2001. Comparison of kinematic and temporal parameters between different pitch velocity groups. J Appl Biomech. 17:1–13.

[cit0012] McClure PW, Michener LA, Sennett BJ, Karduna AR. 2001. Direct 3-dimensional measurement of scapular kinematics during dynamic movements in vivo. J Shoulder Elbow Surg. 10(3):269–277. 11408911 10.1067/mse.2001.112954

[cit0013] Mourtacos SL, Sauers EL, Downar JM. 2003. Adolescent baseball players exhibit differences in shoulder mobility between the throwing and non-throwing shoulder and between divisions of play. J Athl Train. 38(suppl 2):S72.

[cit0014] Myers JB, Laudner KG, Pasquale MR, Bradley JP, Lephart SM. 2005. Scapular position and orientation in throwing athletes. Am J Sports Med. 33(2):263–271. 15701613 10.1177/0363546504268138

[cit0015] Oyama S, Myers JB, Wassinger CA, Ricci RD, Lephart SM. 2008. Asymmetric resting scapular posture in healthy overhead athletes. J Athl Train. 43(6):565. 19030133 10.4085/1062-6050-43.6.565 PMC2582547

[cit0016] Putnam CA. 1993. Sequential motions of body segments in striking and throwing skills: descriptions and explanations. J Biomech. 26(Suppl 1):125–135. 8505347 10.1016/0021-9290(93)90084-r

[cit0017] Ramos E, Frontera WR, Llopart A, Feliciano D. 1998. Muscle strength and hormonal levels in adolescents: gender related differences. Int J Sports Med. 19(8):526-531. 9877143 10.1055/s-2007-971955

[cit0018] Thomas SJ, Swanik KA, Swanik CB, Kelly JD IV. 2010. Internal rotation and scapular position differences: a comparison of collegiate and high school baseball players. J Athl Train. 45(1):44. 20064047 10.4085/1062-6050-45.1.44 PMC2808753

[cit0019] Tsai NT, McClure PW, Karduna AR. 2003. Effects of muscle fatigue on 3-dimensional scapular kinematics. Arch Phys Med Rehabil. 84(7):1000–1005. 12881824 10.1016/s0003-9993(03)00127-8

